# RNA-seq analysis of broiler liver transcriptome reveals novel responses to high ambient temperature

**DOI:** 10.1186/1471-2164-15-1084

**Published:** 2014-12-10

**Authors:** Derrick J Coble, Damarius Fleming, Michael E Persia, Chris M Ashwell, Max F Rothschild, Carl J Schmidt, Susan J Lamont

**Affiliations:** Department of Animal Science, Iowa State University, Ames, IA 50011 USA; Department of Poultry Science, North Carolina State University, Raleigh, NC 27695 USA; Department of Animal and Food Sciences, University of Delaware, Newark, DE 19716 USA

## Abstract

**Background:**

In broilers, high ambient temperature can result in reduced feed consumption, digestive inefficiency, impaired metabolism, and even death. The broiler sector of the U.S. poultry industry incurs approximately $52 million in heat-related losses annually. The objective of this study is to characterize the effects of cyclic high ambient temperature on the transcriptome of a metabolically active organ, the liver. This study provides novel insight into the effects of high ambient temperature on metabolism in broilers, because it is the first reported RNA-seq study to characterize the effect of heat on the transcriptome of a metabolic-related tissue. This information provides a platform for future investigations to further elucidate physiologic responses to high ambient temperature and seek methods to ameliorate the negative impacts of heat.

**Results:**

Transcriptome sequencing of the livers of 8 broiler males using Illumina HiSeq 2000 technology resulted in 138 million, 100-base pair single end reads, yielding a total of 13.8 gigabases of sequence. Forty genes were differentially expressed at a significance level of *P*-value < 0.05 and a fold-change ≥ 2 in response to a week of cyclic high ambient temperature with 27 down-regulated and 13 up-regulated genes. Two gene networks were created from the function-based Ingenuity Pathway Analysis (IPA) of the differentially expressed genes: “Cell Signaling” and “Endocrine System Development and Function”. The gene expression differences in the liver transcriptome of the heat-exposed broilers reflected physiological responses to decrease internal temperature, reduce hyperthermia-induced apoptosis, and promote tissue repair. Additionally, the differential gene expression revealed a physiological response to regulate the perturbed cellular calcium levels that can result from high ambient temperature exposure.

**Conclusions:**

Exposure to cyclic high ambient temperature results in changes at the metabolic, physiologic, and cellular level that can be characterized through RNA-seq analysis of the liver transcriptome of broilers. The findings highlight specific physiologic mechanisms by which broilers reduce the effects of exposure to high ambient temperature. This information provides a foundation for future investigations into the gene networks involved in the broiler stress response and for development of strategies to ameliorate the negative impacts of heat on animal production and welfare.

## Background

Heat-related stress is a key concern for the poultry industry, especially with climate change and the expansion of poultry production into regions of the world that experience extreme temperatures [1]. Heat-related stress occurs when a negative balance exists between the net energy released by a chicken into the environment, and the amount of heat energy produced [2]. This negative balance results in reduced feed consumption, digestion inefficiency, impaired metabolism, and death in broiler chickens [3–5]. St. Pierre et al. [6] estimated the annual heat-related economic loss incurred by broiler, layer, and turkey producers in the U.S. to be approximately $125-165 million [6]. The annual heat-related losses incurred in the broiler sector alone were estimated at $51.8 million [6]. In the past few decades, genetic selection for broiler performance has resulted in remarkable improvements in growth rates [7, 8]. The deleterious effects of high ambient temperature on growth rate are greater in broilers with higher growth rates than those with lower growth rates [9]. Thus, understanding the genetic basis of the physiologic response to heat is critical for improved production efficiency and welfare of poultry.

Few experiments reported to date have investigated the effects of heat on the transcriptome of chicken tissues [10, 11]. Li et al. [10] investigated the transcriptome of broiler breast tissue in response to cyclic high ambient temperature (6-hour daily cycles of 33°C, day 28 to 49 post-hatch) [10]. Of the 110 genes that were differentially expressed in response to high ambient temperature, four (*PM20/PM21*, *ASB2*, *USP45*, and *TFG*) were novel heat-related stress genes. Gene ontology analysis suggested involvement of the mitogen-associated protein kinase (MAPK), ubiquitin-proteasome, and nuclear factor kappa-light-chain-enhancer of activated B cells (*NFKB*) pathways in the response of broilers to high ambient temperature. Exposing L2 Taiwanese roosters to high ambient temperature (4-hour heat stress at 38°C) resulted in the up-regulation of 169 genes and the down-regulation of 140 genes in testis tissue [11]. These genes were primarily involved in response to stress, transport, signal transduction, and metabolism.

The objective of the current study is to characterize the effects of cyclic high ambient temperature on the transcriptome of a highly metabolically active organ in broiler chicks, the liver. This is the first reported study to use RNA-seq to characterize the transcriptome of metabolic-related tissue in broilers exposed to high ambient temperature, thus providing novel insight into the effects of heat on metabolism in broilers. This information provides a platform for future investigations into the gene networks relevant to the production of commercial broilers resilient to the negative impacts of heat.

## Results

### Sequencing the transcriptome, aligning and mapping reads to the genome

Approximately 138 million, 100 base pair single-end reads were generated using Illumina HiSeq 2000 technology to sequence the cDNA libraries. This yielded 13.8 gigabases of total sequence and provided on average 17,249,597 reads per sample. Using the Genomic Short-Read Nucleotide Alignment Program (GSNAP) [12], 83% or more of the reads from each sample mapped back to the reference genome after alignment. Full length (100 bp), single-end reads were used for this analysis to preserve as much sequence information as possible.

### Counting mapped reads

On average, each sample had a total of 11,695,581 uniquely mapped reads. Of these reads, 2,375,095 were classified as “no feature”, meaning that the reads could not be assigned to any feature in the genome. Additionally, 332,309 reads per sample were classified as ambiguous, meaning that the reads were assigned to multiple genomic features.

### Testing for differential expression and pathway analysis

Forty genes were differentially expressed at a significance level of *P*-value < 0.05 and a fold-change ≥ 2 in response to cyclic high ambient temperature. Of these 40 genes, 27 were down-regulated and 13 up-regulated. The fold-changes induced by high ambient temperature ranged from -12.5 to 20.0 (Table 1). Two gene networks were created from the Ingenuity Pathway Analysis (IPA) of these genes: “Cell Signaling” and “Endocrine System Development and Function”.

**Table 1:** **Significance levels of differentially expressed hepatic genes in response to high ambient temperature**

Gene	***P***-value	Fold change	Q-value
RFX6	0.01	-12	0.6
LOC100857	0.007	-11	0.6
SPSB4	0.001	-11	0.3
GPR133	0.0005	-11	0.2
TRPC5	0.02	-11	0.7
LOC427426	0.02	-11	0.7
BNC1	0.004	-11	0.5
LOC423425	0.02	-10	0.7
ANGPTL4	0.0006	-3	0.2
LOC100859	0.03	-2	0.7
LOC420770	0.04	-2	0.7
LOC100858	0.01	-2	0.7
KRT14	0.009	-2	0.6
FMOD	0.001	-2	0.3
LOC419500	0.004	-2	0.5
LOC100858	0.01	-2	0.7
SCN3B	0.03	-2	0.7
S100A4	0.005	-2	0.5
LOC100858	0.04	-2	0.7
TRIM50	0.05	-2	0.7
BRCC3	0.02	-2	0.7
LOC771141	0.08	-2	0.7
PDGFD	0.03	-2	0.7
AKR1C3	0.05	-2	0.7
SPON1	0.03	-2	0.7
DIO2	0.006	-2	0.5
NID1	0.01	2	0.6
HFM1	0.01	2	0.6
BDKRB1	0.04	2	0.7
CCK	0.04	2	0.7
MYRIP	0.007	2	0.5
ORMDL3	0.05	2	0.7
RNF220	0.03	2	0.7
LIMS2	0.02	2	0.7
ERC2	0.02	2	0.7
S100A1	0.005	2	0.5
FGF14	0.008	2	0.5
DIO3	0.00009	3	0.1
LOC395159	0.001	5	0.3
LOC100857	0.0008	20	0.3

**Figure 1:**
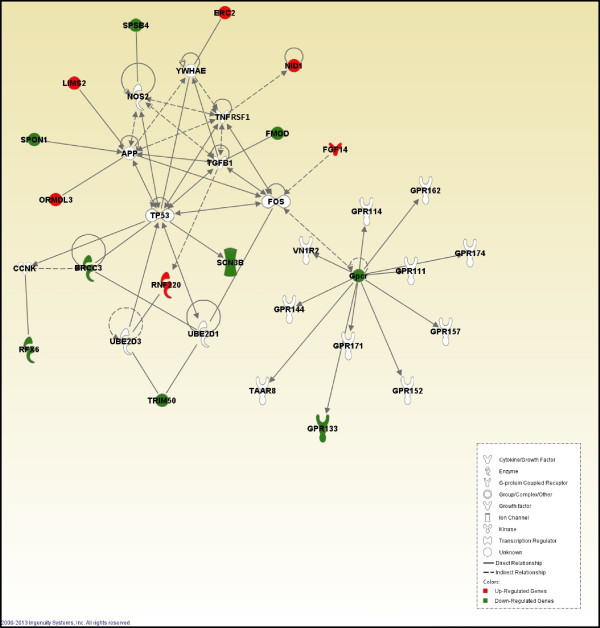
**Cell signaling.** Pathway analysis of gene functions in broiler liver transcriptome in response to cyclic high ambient temperature. Red color shows up-regulation and green color shows down-regulation (IPA). White molecules are not differentially expressed, but are included to illustrate associations with significantly up- and down-regulated genes.

The differentially expressed gene products in the “Cell Signaling” network included *BRCA1/BRCA2*-containing complex, subunit 3 (*BRCC3*), *ELKS*/*RAB6*-interacting/CAST family member 2 (*ERC2*) isoform X1, fibroblast growth factor 14 (*FGF14*), *FMOD* (fibromodulin), G protein-coupled receptor 133 (*GPR133*), *LIM* and senescent cell antigen-like domains 2 (*LIMS2* ) isoform X2, nidogen-1 (*NID1*), *ORM1*-like 3 (*Saccharomyces cerevisiae*) (*ORMDL3*), regulatory factor x-box binding family transcription factor member 6 (*RFX6* ) isoform X2, ring finger protein 220 (*RNF220*), sodium channel, voltage-gated, type III, beta subunit (*SCN3B*), spondin 1, extracellular matrix protein (*SPON1*), splA/ryanodine receptor domain and SOCS box containing 4 (*SPSB4*) isoform X3, and tripartite motif containing 50 (*TRIM50*) (Figure 1).

**Figure 2:**
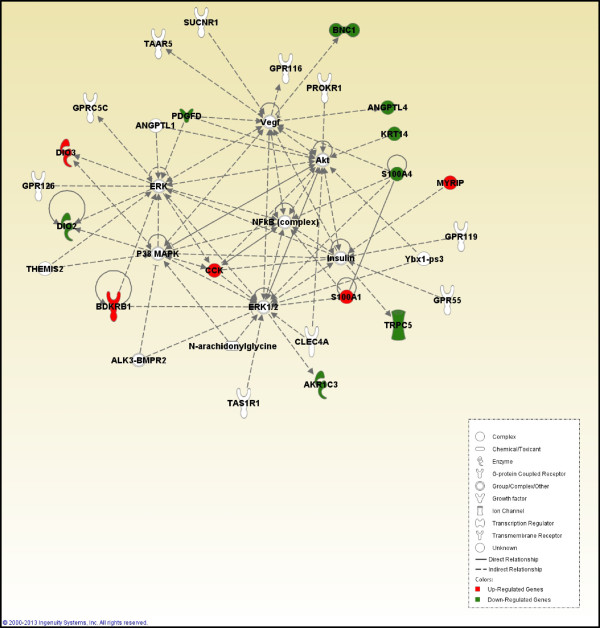
**Endocrine system development and function.** Pathway analysis of gene functions in broiler liver transcriptome in response to cyclic high ambient temperature. Red color shows up-regulation and green color shows down-regulation (IPA). White molecules are not differentially expressed, but are included to illustrate associations with significantly up- and down-regulated genes.

**Figure 3:**
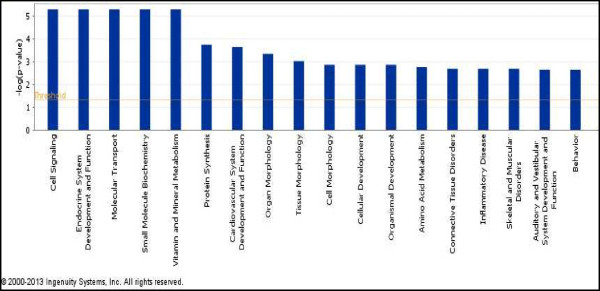
**Significance levels of functions of differentially expressed hepatic genes in response to high ambient temperature.** Threshold was set at *P* = 0.05 and indicated as -log (*P*-value) on the Y-axis. X-axis shows functions of differentially expressed genes.

The “Endocrine System Development and Function” network included aldo-keto reductase family 1, member C3 (*AKR1C3*), angiopoietin-like 4 (*ANGPTL4* ), bradykinin receptor B1 (*BDKRB1*), basonuclin 1 (*BNC1*) isoform X4, cholecystokinin (*CCK*), deiodinase, iodothyronine, type II (*DIO2*), deiodinase, iodothyronine, type III (*DIO3*), keratin 14 (*KRT14*), myosin VIIA and Rab interacting protein (*MYRIP*), platelet derived growth factor D (*PDGFD*), *S100* calcium binding protein A1 (*S100A1*), *S100* calcium binding protein A4 (*S100A4* ), and transient receptor potential cation channel, subfamily C, member 5 (*TRPC5*) (Figure 2).To determine the biological pathways elicited in the liver by exposure of the birds to heat, the differentially expressed genes were categorized by function. Cell signaling, endocrine system development and function, molecular transport, small molecule biochemistry, and vitamin and mineral metabolism were the most significant functions elicited in response to heat (Figure 3).

### qPCR (quantitative polymerase chain reaction)

Five up-regulated and 4 down-regulated genes were selected for qPCR analysis (*P*-value ≤ 0.05, fold-change ≥ 2) (Table 2). All samples represented in the RNA-seq analysis, plus samples from 8 additional broilers exposed to the same treatments were included in the qPCR analysis, resulting in 8 samples per treatment. Data from 8 of the 9 genes were in concordance between RNA-seq and qPCR analyses. For 4 genes, the qPCR differential expression between groups was in agreement with the results of the RNA-seq, and was significant. For an additional 4 genes, the relative ranking of the treatment means was the same for both qPCR and RNA-seq, but the differences were not significant for qPCR. There was discordance between RNA-seq and qPCR in the relative expression levels between the temperature treatments for only one of the nine genes (*LIMS2*).

**Table 2:** ***P-*** **values from RNA-seq and qPCR validation**

Gene	RNA-seq	qPCR
SPSB4	0.001	0.008
TRPC5	0.02	0.2
BNC1	0.004	0.1
ANGPTL4	0.0006	0.02
LIMS2	0.02	0.4
ERC2	0.02	0.8
S100A1	0.005	0.008
FGF14	0.008	0.7
DIO3	0.00009	0.02

## Discussion

The number of differentially expressed genes detected in the current study (n = 40) is not high, which may be because of several factors. Applying thresholds of both significance level and a minimum fold-change may have reduced the total number of genes that were declared as differentially expressed. Using four replicates of full-sib pairs of heat-exposed and control chickens reduced random genetic variation between treatment groups, and therefore may have reduced the number of differentially expressed genes occurring at random. The low number of detected differentially expressed genes is not, however, likely to be attributable to the animal number because the sample number used in this experiment is comparable to other RNA-seq experiments. Wolf and Bryk [13] used four replicates of male and female chickens to study dosage compensation in chickens using RNA-seq analysis; Ayers et al. [14] used two male and two female chickens to incorporate RNA-seq analysis to examine the sexual dimorphic gene expression before gonadal differentiation. The method used to estimate the data variance determines the level of detection of differential expression in RNA-seq data [15]. The current study has increased power to determine differential expression because of the advantages of the QuasiSeq package over other methods, such as DESeq and EdgeR.

The differential expression of two genes (*BRCC3* and *FGF14*) from the “Cell Signaling” network and five genes (*CCK*, *TRPC5*, *DIO2*, *DIO3*, and *ANGPTL4*) from the “Endocrine System Development and Function” network indicate that birds use specific physiologic mechanisms to regulate their internal temperature in response to high ambient temperature. *CCK*, *DIO3*, *BRCC3*, and *FGF14* were up-regulated, while *TRPC5*, *DIO2*, and *ANGPTL4* were down-regulated. CCK inhibits feed intake in chickens by promoting gastric emptying, stimulating the release of pancreatic digestive enzymes, and signaling the brainstem to depress appetite [16–18]. During exposure to heat, animals experience a negative balance in the amount of heat energy released and produced [2]. The inhibition of feed intake may be a mechanism to reduce the additional heat that is produced from digestive metabolism. Although the intestines are responsible for most of the body’s CCK production, *CCK* mRNA expression has also been detected in the liver [19]. Wang et al. [20] suggested that transient receptor potential channels (TRPC) are responsible for cellular CCK signaling. The differential expression of *TRPC5* supports TRPC involvement in CCK signaling. *DIO2* and *DIO3* encode for members of an enzyme family, deiodinases, which is involved in thyroid hormone regulation [21]. Silva [22] demonstrated that thyroid hormone is involved in aerobic metabolism and thermal control. *DIO2* is involved in the preservation of thyroid hormone [23], while *DIO3* is associated with the inactivation of thyroid hormone [24]. Taken together, the contrasting regulation of *DIO2* and *DIO3* is predicted to decrease thyroid hormone levels. Past studies have shown increases in hepatic *DIO3* mRNA expression and activity levels in broilers after feed removal [25–27]. The expression patterns of *CCK*, *TRPC5*, *DIO2* and *DIO3* observed in the current study highlight a direct interaction between feed intake, deiodinase activity, and temperature regulation in response to the heat.

Exposure to high ambient temperature results in a redistribution of blood flow from internal organs to peripheral tissues, reducing the internal temperature of chickens [28]. *BRCC3*, *FGF14*, and *ANGPTL4* are all involved in regulation of blood vessel development [29–31]. Their differential expression, therefore, may be a host response to modulate internal temperature by regulating blood capillary development and distribution.

The differential expression of three genes (*SCN3B*, *SPON1*, and *ORDML3*) from the “Cell Signaling” network and *MYRIP* from the Endocrine System Development and Function” network reflects a response to hyperthermia-induced apoptosis. *SCN3B*, *SPON1*, and *MYRIP* were down-regulated, while *ORDML3* was up-regulated in response to heat. *SCN3B* has been identified as a *TP53*-inducible proapoptotic gene in mouse embryonic cells [32]. The down-regulation of *SCN3B* reduces the capability of apoptosis through the *TP53* pathway. *SPON1* encodes for a secreted adhesion molecule that attaches to the extracellular matrix (ECM) of cells and inhibits amyloid β (A4) precursor protein (APP) cleavage into β-secretase [33–35]. The cleavage of APP results in amyloid β fibril formation, and induces apoptosis in yeast through mitochondrial dysfunction [36, 37]. In the current study, the down-regulation of *SPON1* may be a mechanism to return to homeostatic *SPON1* mRNA levels following a transient up-regulation. Haughey et al. [38] suggested that amyloid β fibril accumulation occurs within the lipid rafts, which are specialized membrane domains mainly consisting of sphingolipids and cholesterol. *ORMDL3* belongs to a family of genes (ORM) that has been implicated in sphingolipid homeostasis [39]. In yeast, heat results in a transient accumulation of sphingolipid that can lead to apoptosis through sphingolipid signaling [40, 41]. The up-regulation of *ORMDL3* reflects a cellular mechanism to regulate sphingolipid levels in response to heat, thus preventing apoptosis. *MYRIP* encodes for an actin motor that drives vesicle and organelle motility, and participates in endosome recycling [42]. The up-regulation of *MYRIP* may lead to enhanced ability of phagocytic cells to engulf and recycle the cellular particles after hyperthermia-induced apoptosis.

The down-regulation of three genes (*FMOD*, *GPR133*, and *TRIM50*) from the “Cell Signaling” network reflects the latter stages of a host-regulated tissue repair response. As a liver proteoglycan, FMOD regulates ECM organization by fibrogenic stimuli in mouse liver cells and has been described as essential for tissue repair [43]. Hepatic fibrogenesis results from damage to hepatocytes by inflammatory reactions, which alter their ECM [44]. Ruddell et al. [45] suggested that hepatic fibrogenesis was enhanced by cross-talk between hepatic lipocytes and hepatic progenitor cells (HPC), resulting in chemotaxis and cell migration. As an adhesion G protein-coupled receptor (GPR), *GPR133* is thought to participate in cell-to-cell and cell-matrix interactions [46, 47]. *TRIM50* participates in inflammation suppression, fibroblast migration, and lymphocyte chemotaxis in mouse embryo fibroblasts [48, 49].

The up-regulation of *ERC2* from the “Cell Signaling” network and the differential expression of three genes (*S100A1*, *S100A4*, and *BDKRB1*) from the “Endocrine System Development and Function” network reflect a disruption in hepatocyte calcium levels in response to heat. *ERC2*, *S100A1*, and *BDKRB1* were all up-regulated, while *S100A4* was down-regulated. *ERC2* encodes for a protein that localizes at voltage dependent calcium channels (VDCC) [50]. The S100 family of proteins function in the regulation of calcium homeostasis, cell growth and differentiation, protein phosphorylation, and the inflammatory response [51]. The differential expression of an S100 family member (*S100A11*) and other genes involved in calcium-related transport and signaling was observed in catfish exposed to high ambient temperature [52]. Texel and Mattson [53] reported that excess amyloid β fibril formation perturbed calcium homeostasis in mouse neuronal cells, possibly triggering apoptosis from calcium overload. *BDKRB1* encodes for a bradykinin receptor that belongs to the rhodopsin-like GPRs that function in the regulation of inflammation [54]. The activation of *BDKRB1* increases cytosolic calcium levels [55]. The up-regulation of *BDKRB1* is a component of an inflammatory response that contributes to perturbed cellular calcium levels. The up-regulation of *ERC2* and *S100A1* represents calcium transport through calcium channels and calcium-dependent cellular signaling. The down-regulation of *S100A4* may compensate for the calcium-related transport and signaling that is carried out by the proteins encoded by *ERC2* and *S100A1*. The differential expression of *ERC2*, *S100A1*, and *S100A4* illustrates the dynamic mechanisms that regulate cellular calcium levels in response to heat.

In summary, the liver transcriptome of broilers exposed to high ambient temperature indicates specific host responses to decrease internal temperature, reduce hyperthermia-induced apoptosis, and promote tissue repair. Additionally, differential expression occurred in genes that regulate the perturbed cellular calcium levels that result from exposure to heat.

## Conclusions

Cyclic high ambient temperature causes metabolic, physiologic, and cellular-level changes that can be characterized through RNA-seq analysis of the liver transcriptome. The current findings suggest that these changes induce specific mechanisms by which broilers can reduce the negative physiologic effects of heat exposure. These novel insights into the effects of high ambient temperature on the metabolic transcriptome of broilers provide a foundation for future investigations into the gene networks involved in the response to heat, and for development of strategies to ameliorate the negative impacts of hot climates on animal welfare and productivity.

## Methods

### Tissue collection

From 22 to 28 days of age, heat-treated broilers were exposed to daily 7-hour cycles of 35°C, while a control group was kept at 25°C throughout this time. The experiment was replicated with birds from different hatches. Liver samples were harvested from 4 sets of full-sibs at the midpoint of the last heat cycle on day 28, with one bird of each pair from the high ambient temperature treatment and one from the control temperature group; these 8 samples were used for transcriptome sequencing. Liver samples were also harvested from 8 additional broilers (4 control and 4 heat-treated) from the same study, and included with the 8 samples used for RNA-seq for the qPCR validation of the transcriptome sequencing. All animal experiments were approved by the Iowa State University Institutional Animal Care and Use Committee: Log #4-11-7128-G.

### Sequencing the transcriptome

Total RNA was isolated from the liver samples following previously described procedures [56]. The RNA quality was assessed using an Agilent 2000 Bioanalyzer. Seven was the threshold RNA Integrity Number (RIN) score for cDNA library construction. The RNA was submitted to the Iowa State University DNA Facility, where libraries from each of the 8 individuals were generated and the individual liver transcriptomes were sequenced using Illumina HiSeq 2000 technology (http://www.illumina.com/systems/hiseq_2000_1000.ilmn). All 8 samples were sequenced on one lane.

### Quality control of RNA-seq reads

Read quality was controlled using the FastQC suite version 0.10.1. (https://preview.iplantcollaborative.org/de/#workspace). A Phred score of 28 was used to control for read quality. The adaptor sequences were removed from the reads using the fastx_clipper software of the fastx_toolkit (version 0.0.13; http://hannonlab.cshl.edu/fastx_toolkit/).

### Mapping reads to genome

The RNA-seq reads were aligned using GSNAP [12], a program that can be used to align single- or paired-end reads. The authors chose to employ GSNAP over other programs because of the sequencing platform used and the advantages of using GSNAP to map RNA-Seq reads. In the current study, Illumina HiSeq 2000 technology was used to perform sequencing, resulting in 138 million, 100 bp reads. Both Illumina platforms, HiSeq 2000 and Genome Analyzer, use sequencing-by-synthesis (SBS) technology, but HiSeq 2000 has a two- to five-fold higher rate of data acquisition [57]. The ability to detect exon-intron boundaries and the connections between exons is very important when assigning RNA-Seq reads to their molecules of origin. This requires the use of programs called spliced-mappers. These programs are splice-site aware and incorporate intron-like gaps. GSNAP is a splice-mapper that uses a seed-and-extend method that maps part of the gene as substrings, and then uses algorithms to extend candidate matches and locate potential splice sites [58]. This method tends to be less dependent on coverage and more likely to recover novel splice sites. The compatibility of GSNAP to the sequencing platform, the good balance between computing speed and accuracy, the availability of a reference genome, and the wide acceptance of the method are all reasons for which the authors chose GSNAP for mapping the RNA-Seq reads in the current study.

The RNA-seq reads were mapped back to the genome based on the NCBI *Gallus gallus* Build 4.0 reference genome. The data were run using “32 worker threads” to optimize computational efficiency. The output was set to “split” and put into a SAM format for convenient downstream analysis. During the alignment, the “-m” setting for mismatches was set at default to allow GSNAP to auto set the number of allowed mismatches based on read length, which allowed for the best alignment across intron-exon boundaries GSNAP “soft-trims” reads during the alignment process, so that only portions of the reads above the quality threshold are aligned. Table 3 details the number of mapped and non-mapped reads, along with the number of reads before and after FastQC quality filtering (Phred 28).

**Table 3:** **Number of reads before and after FastQC filtering, and number of mapped and non-mapped reads**

cDNA library	Reads (Pre-filter)	Reads (Post-filter)	Mapped reads	Non-mapped reads
2145	14,518,971	14,102,129	10,786,983	3,315,146
2146	16,435,957	16,079,777	11,966,390	4,113,387
2153	16,143,847	15,893,619	10,958,652	4,934,967
2154	12,443,771	12,157,917	9,162,608	2,995,309
2162	13,121,981	12,695,265	8,949,977	3,745,288
2163	19,905,505	19,516,053	14,835,601	4,680,452
2169	22,161,153	21,786,920	15,456,927	6,329,993
2170	15,633,924	15,633,924	11,468,175	4,165,749
**Total**	130,365,109	127,865,604	93,585,313	34,280,291
**Standard error**	±816.15	±820.30	±245.41	±179.13

### Counting mapped reads

Raw reads were calculated and annotated using the HT-seq package (version 4.7) in Python (http://www-huber.embl.de/users/anders/HTSeq/), which is an open source program that allows the input of raw counts from aligned reads to be annotated with gene names based on genomic features. The parameters “m < mode>”, and “--mode = <mode > =” were set to “intersection non-empty”. The stringency of these settings allows HT-seq to identify as many genes as possible based on overlap resolution. The parameter “-stranded” was set to “no” because the cDNA library preparation was not strand specific. The parameter “–a < minaqual” was set to “default (0)” because the read quality threshold was cutoff at Phred 28. The feature type was set at “-- type = exon” so that the reads would be counted based on exons. The GFF attribute used as the feature ID (“--idattr = <id attribute>”) was set to “gene_id”, allowing the NCBI GFF file to output gene names. For unmapped reads, special categories were provided that explained why the reads were not mapped back to the reference genome.

### Testing for differential expression

To account for the over-dispersion associated with RNA-seq data, the QuasiSeq [59] package developed in R [60] was used to analyze the data for differential expression. The QuasiSeq package was chosen for differential expression analysis because of its advantages over DESeq in detecting differentially expressed genes. Upon estimating negative binomial dispersion parameters, DESeq treats the resulting estimates as constants, which can result in a non-uniform distribution of *P*-values and inaccurately estimates false discovery rates [59]. When estimating dispersions, the QuasiSeq package allows gene-specific estimates to vary around a central estimated trend and shares information across genes. When testing for differential expression, the QuasiSeq package employs a quasi-likelihood method that incorporates uncertainty in the estimated variances and provides a self-tuning approach to shrinking gene-specific dispersion estimates [59]. Differential expression was declared at a significance level of *P*-value < 0.05 and a fold-change ≥ 2.

### Gene network analysis

Upon selecting *Gallus gallus* in the settings, gene networks were constructed using Ingenuity Pathways Analysis [61]. Statistically significant networks were considered with a *P*-value cut-off of 0.0001. This analysis was used to identify gene interactions within these networks.

### qPCR primer design

The full cDNA sequences for each gene were obtained from NCBI. The cDNA sequences were uploaded into the NCBI Splign database and *Gallus gallus* was selected as the reference genome. This allowed the authors to view only the exonic regions of each gene. Sequences of 20–22 nucleotides (nt) were chosen for forward primers. These sequences were in 5’-3’ (sense) orientation to the cDNA sequences and spanned exon junctions. Along with the NCBI RefSeq numbers for each gene; these forward sequences were input into Primer3, a primer design program. Primer3 is open-source software that has been widely used for primer design for over a decade [62]. It was used to select reverse sequences that were in 3’-5’ (antisense) orientation to the cDNA sequences and also spanned two exons. Using primers that span exon junctions prevents the amplification of genomic DNA [63]. Primer3 allowed assessment of the quality of the primer set (melting temperature, GC%, and self-complementarity). The output also included the product size, the sequence in FASTA format, and where the primers will anneal to the sequence. Primer3 thresholds were set to ensure a product size of 60–150 nt, a GC content of 50-60%, a primer length of 18–24 nt, and a melting temperature of 60-63°F. Primer sets that didn’t span exon junctions, contained primers that were too complementary to one another, or didn’t meet these specifications were rejected. Sequencing and NCBI Primer-BLAST, which checks the specificity of a primer set with all possible regions of the genome, were used for primer target detection.

### qPCR and statistical analysis

Total RNA was isolated and quantitative PCR was performed according to previously described procedures [56]. All reactions were run in triplicate. The forward and reverse primers used for qPCR are listed in Table 4. The amplicons were verified for specificity and reaction quality using the melting curves of the reactions. The cycle threshold (Ct) line was adjusted to fit the standard curve with an acceptable r^2^ value, 0.96-1. The efficiency of each primer set was calculated using the following formula: 10^[(-1/slope)-1] × 100. Table 5 shows the r^2^ values, efficiencies, and NCBI RefSeq number for each primer set, along with the product length and the length of each primer. Adjusted Ct values for statistical analysis were calculated as follows: 40 - [(sample mean Ct) + (median 28S Ct – mean 28S Ct) × (sample gene slope/28S slope)] [64]. The mRNA expression levels as mean adjusted Ct values of each triplicate sample were analyzed using the ANOVA analysis of JMP 8.0.2 software on combined data (control and temperature-exposed broilers) for each gene separately, using the following model:

**Table 4:** **Forward and reverse primers used for qPCR validation of RNA-seq**

Gene	Forward primer	Reverse primer
SPSB4	5′ AATGGACTTGACCCGGAAC 3′	5′ TTTCAGAGACAGAGGCAAAGG 3′
TRPC5	5′ CTGCCCTGGGTTCTAGGTTT 3′	5′ GGGAGTTCATTGCAAAATCC 3′
BNC1	5′ TGGATATGTGCTGCAGGATG 3′	5′ TGCCATTAACTCCACAATGG 3′
ANGPTL4	5′ TGTGACATGACTGCAGAAGG 3′	5′ CAGCCAGAAGTCACCATGAA 3′
LIMS2	5′ CAGATGGGCTTTTCTATGAGTT 3′	5′ GAAACATTCTGGGTGCCAGT 3′
ERC2	5′ GTCTTGCCTCAACACAGCAA 3′	5′ GGCAATGTTTGCATCCTTTT 3′
S100A1	5′ AGCTGAGCAAGAAGGAGCTG 3′	5′ GGTCCTGCATGATCTTCTCC 3′
FGF14	5′ TACCCAAGCCATTGGAAGTT 3′	5′ GTTTGCCGCCATTCATTATT 3′
DIO3	5′ GCTCTCTTCCTTCGGGATCT 3′	5′ CCCATTTCAAAATCGGTCAT 3′

**Table 5:** **Primer set r** ^**2**^ **values and efficiencies, along with the length of each amplicon and primer**

Primer	Length	Product size	r ^2^	Efficiency
SPSB4 For	20 bp	120 bp	1	100%
SPSB4 Rev	21 bp			
TRPC5 For	20 bp	112 bp	1	104%
TRPC5 Rev	20 bp			
BNC1 For	20 bp	133 bp	1	109%
BNC1 Rev	20 bp			
ANGPTL4 For	20 bp	126 bp	1	91%
ANGPTL4 Rev	20 bp			
LIMS2 For	20 bp	143 bp	1	100%
LIMS2 Rev	20 bp			
ERC2 For	20 bp	149 bp	1	104%
ERC2 Rev	20 bp			
S100A1 For	20 bp	120 bp	1	95%
S100A1 Rev	20 bp			
FGF14 For	20 bp	129 bp	1	109%
FGF14 Rev	20 bp			
DIO3 For	20 bp	143 bp	1	104%
DIO3 Rev	20 bp			

Challenge and replicate were considered fixed effects. Student’s t test of JMP 8.0.2 software was used to determine significant differences (*P*-value < 0.05) between high ambient temperature and control treatments.

### Data deposition

The data discussed in this publication have been deposited in NCBI's Gene Expression Omnibus [65] and are accessible through GEO Series accession number GSE51035 (http://www.ncbi.nlm.nih.gov/geo/query/acc.cgi?acc=GSE51035).
